# Infrastructure collapsed, health care access disrupted, Myanmar people with chronic diseases are in danger

**DOI:** 10.7189/jogh.13.03002

**Published:** 2023-01-11

**Authors:** Wei-Ti Chen, Chengshi Shiu, Franco R Lee, Saiyud Moolphate, Myo Nyein Aung

**Affiliations:** 1 School of Nursing, University of California Los Angeles, Los Angeles, California, USA; 2 School of Social Work, National Taiwan University, Taipei, Taiwan; 3 Palos Verdes Peninsula High School, Palos Verdes, California, USA; 4 Department of Public Health, Chiangmai Rajabhat University, Chiangmai, Thailand; 5 Faculty of International Liberal Arts and Department of Global Health Research, Graduate School of Medicine, Juntendo University, Tokyo, Japan

The situation in Myanmar is getting worse. As civil war and military strikes erupt in many locations inside Myanmar, cities in nearby countries such as Thailand, Bangladesh, and India, report more wounded and internally displaced people (IDP) from Myanmar in their local clinics and hospitals [1]. Many IDP came both from nearby regions of Myanmar and distant major cities (such as Yangon and Mendeley) and travelled large distances to reach the border. These migrations to border countries are not only driven by repressive military rule and civil unrest, but also by financial strain, food insecurities and limited access to health care supplies and treatments.

After almost two years under the military junta regime, people in Myanmar struggle to live amid economic and political turmoil. According to 2022 World Bank data, 40% of Myanmar’s population lives below the poverty line, reversing years’ worth of efforts in poverty reduction [2]. In October 2022, the Financial Action Task Force (FATF) added Myanmar to their blacklist, together with Iran and North Korea, for money laundering and terrorism financing, leading to a dramatic depreciation of Myanmar’s currency [3]. A rapid depreciation of the Kyat against the dollar means goods are now more expensive, and the banking system has placed limits on daily withdrawals to US$1000, making life even more difficult for people already struggling to survive [4]. Increased global oil prices causing spikes in domestic fuel prices and transportation costs have translated into dramatic price hikes in diesel generator operation costs, resulting in regular electricity outages. As the ongoing internal conflicts continue to erode infrastructures in Myanmar, currency depreciation, higher transportation costs and the withdrawal of foreign investments have all resulted in declining household incomes and unemployment and have given rise to higher levels of poverty and food insecurity.

Even before the takeover by the military, junta the Myanmar health infrastructure reported long-standing inequalities and lack of visible health equity [4]. Previously, local clinics and hospitals could oversee people with health care needs. Now, health care infrastructures have all but collapsed, with many health care providers participating in civil disobedience movements (CDM). Universities are closed both due to the pandemic and because of faculty shortages within clinical departments caused by their participation in CDM [5]. After the coup, medicine and health care-related supplies increased three to four times [4]. Now, people need to seek help and pick up daily medications (e.g. hypertension, diabetes and/or human immunodeficiency virus (HIV) drugs) in nearby towns. If nearby clinics are out of medicine, patients usually need to travel further distances, only to pay even higher prices for their medications. However, due to lack of gasoline and more casualties, travelling to distant sites is not always possible. For those who require chemotherapy or operations, private hospitals charge in foreign currency for their services. For example, foreign-based hospitals will deny even scheduling any treatment or procedures until funds can be secured by their facilities [6]. This climate has driven many Burmese to relocate to neighboring countries for their health care and lead those who cannot travel to disengage from their health care.

Recent reports on military attacks on IDP include confronting market gatherings, villages, pagodas, schools, and other midway transiting sites and amounts to deliberately blocking all humanitarian aid to IDP [7]. Aid includes personal protection equipment, medical supplies, medicines, and even food. Aid blockages have increased the potential for infections and the negative impact of infectious and chronic diseases, e.g. COVID-19, hypertension, diabetes, and HIV [8]. Myanmar’s mobile population and IDP face both stigmatization from their host countries and health care access deprivation due to their undocumented status [9]. Migrants in nearby countries struggle with a lack of health care access in the host country, are last to receive COVID-19 vaccinations, are exposed to unstable, unsanitary, and unsecure living and working environments, and experience mental stress, acculturation, and culture shock [10].

**Figure:**
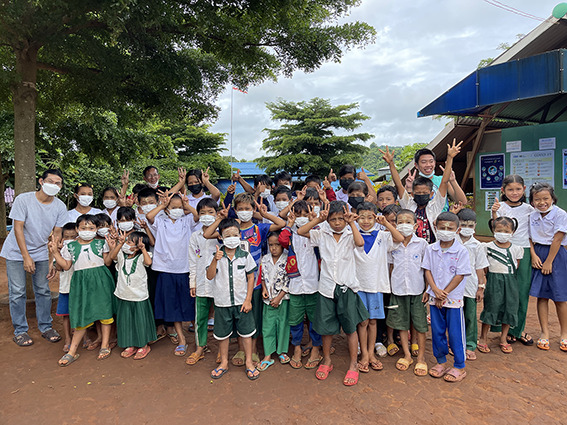
Photo: The tallest three people are W. Zaw (far left), the school principal (middle), F. Lee (far right), with Myanmar IDP children in Mae Sot, Thailand. Source: Wei-Ti Chen’s own collection. Used with permission from the Global Action.

During a recent UN assembly, UN-appointed independent human rights expert Tom Andrews stated that “1.3 million Myanmar people are displaced; 28 000 destroyed homes; villages burned to the ground; more than 13 000 children killed as the death toll for innocent people rises significantly; a looming food crisis; and 130 000 Rohingya in de facto internment camps” [9]. These statistics describe the humanitarian crisis that has become synonymous with Myanmar. Attention on Myanmar from global community is urgently needed to address the ongoing humanitarian crisis. International pressure is required to cease the bloodshed and suffering inside the country and provide health care to all people in need, which constitutes a basic human right. Working with local and international NGOs can be the first step to understanding the population’s needs, so that even rudimentary care for IDP in Myanmar borders can be provided.
